# Synthesis and Characterization of Sm^3+^/Yb^3+^ Codoped Oxychloride Tellurite Glasses for Solar Cell Enhancement via Energy Conversion

**DOI:** 10.1021/acsomega.5c03579

**Published:** 2025-07-18

**Authors:** Gleice A. C. Pinto, Fábio A. dos Santos, Sandro M. Lima, Luis H. C. Andrade, Junior R. Silva

**Affiliations:** † Grupo de Síntese e Estudo de Materiais Fotônicos—GSEMF, Centro de Estudos em Recursos Naturais—CERNA, 67708 Universidade Estadual de Mato Grosso do Sul—UEMS, Dourados, Mato Grosso do Sul 79804-970, Brazil; ‡ Grupo de Pesquisa de Materiais Fotônicos e Energia Renovável, 186079 Universidade Federal da Grande Dourados, Dourados, Mato Grosso do Sul 79804-970, Brazil; § Grupo de Espectroscopia Óptica e Fototérmica—GEOF, Centro de Estudos em Recursos Naturais—CERNA, Universidade Estadual de Mato Grosso do Sul—UEMS, Dourados, Mato Grosso do Sul 79804-970, Brazil

## Abstract

This study presents the synthesis and characterization of novel oxychloride tellurite glasses codoped with Sm^3+^/Yb^3+^. These materials were investigated for their potential applications as spectral converters to enhance the efficiency of silica-based solar cells. Optical spectroscopy revealed the successful incorporation of both rare-earth ions in the glass matrix. It was observed that with the increase of Yb^3+^ ion concentration, the lifetime of the ^4^G_5/2_ state of Sm^3+^ ions decreased considerably, indicating an efficient energy transfer mechanism between the Sm^3+^/Yb^3+^ pair. A notable energy transfer efficiency of 76% was achieved for the sample containing 3 mol % of YbCl_3_. The observed visible-to-near-infrared spectral conversion properties make these glasses promising candidates for improving the performance of silicon solar cells by better matching the solar spectrum to the optimal response range of a photovoltaic device.

## Graphical abstract

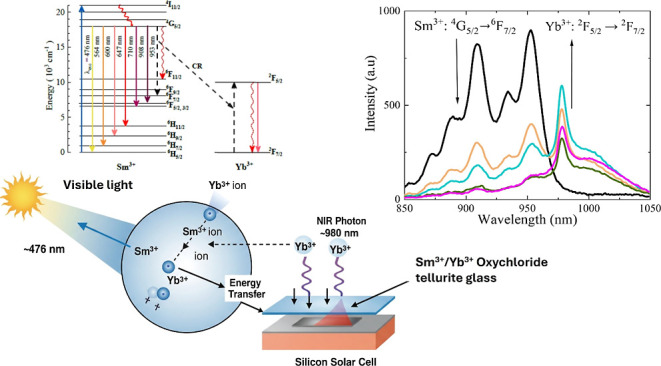

## 1 Introduction

The imperative of addressing global energy demands while advancing sustainable energy production has intensified research efforts toward novel energy sources and conversion technologies. Among renewable alternatives, solar energy demonstrates exceptional potential as a viable long-term solution within the global energy portfolio, particularly through photovoltaic conversion mechanisms that enable direct transformation of solar radiation into electrical power via semiconductor-based solar cells.^1^

Current research aims to increase the efficiency of solar panels, since crystalline silicon (c-Si) solar cells present only 26.7%, very close to the intrinsic limit of around 29%.^2^ The problem leading to this relatively low efficiency is related to the mismatch between the maximum solar intensity spectrum (visible region) and the maximum response of silicon solar cells in the near-infrared region.^3^ It is well-known that the spectral distribution of solar light consists of photons ranging from the ultraviolet to the infrared (280–2500 nm) region, but the panels utilize only a relatively small fraction of this spectrum in the near-infrared to generate electric energy.^4^ This characteristic induces losses due to thermalization and nonabsorption (transmission) in the semiconductor, so that different researchers around the world are looking for alternatives to minimize those drawbacks.

Luminescent materials have been considered as spectral converters to couple in Si solar cells.^5^ Rare-earth ion-doped optical materials can be used through upconversion and downconversion processes. Upconversion involves the absorption of two or more lower-energy photons in the near-infrared region, followed by the emission of a higher-energy photon.^6^ Downconversion absorbs high-energy photons and converts them into lower-energy ones.^7^ In some cases, two lanthanide ions are used to codope the material, in which one of them is used as a sensitizer and the other is the activator. The sensitizer absorbs the photon in low or high energy (for up- or downconversion, respectively) and transfers its energy to the activator that has high luminescence quantum efficiency in the near-infrared region at the maximum response of the Si solar cell.

Materials doped with Yb^3+^ and codoped with Tb^3+^, Tm^3+^, Pr^3+^, Er^3+^, Nd^3+^, and Cr^3+^ ions have been studied for the improvement of Si photovoltaic systems, as they present efficient energy transfer through a downconversion process.^8–10^ In these studies, the Yb^3+^ ion has been used as a good activator ion due to its emission band close to 1000 nm, and in many materials, this ion exhibits high luminescence quantum efficiency, which is a crucial characteristic for an energy converter for Si solar cells.^11^

Tellurite glasses are widely used as hosts for energy conversion due to their optical characteristics such as a wide transmission window, high refractive index, and good solubility of rare earth ions, and when compared to other classes of glass, such as silicates, they have the lowest phonon energy, around 800 cm^–1^, as well as good thermal and chemical stability.^12^ Figueredo et al. (2015) studied the 80TeO_2_–10Li_2_O–10TiO_2_ matrix codoped with Er^3+^/Yb^3+^ and observed the occurrence of energy transfer through a cross-relaxation process, with a maximum efficiency of 56%.^13^ Costa et al. (2017) investigated the energy transfer between Nd^3+^/Yb^3+^ ions in a TeO_2_–WO_3_ matrix. They observed a reduction in the infrared luminescence of Nd^3+^ ions concomitant with an increase in the emission of Yb^3+^ ions, and a high energy transfer efficiency was observed between these ions (96%).^8^ Another study investigated TeO_2_–ZnO glass, codoped with Tb^3+^/Yb^3+^. In this study, it was reported a downconversion mechanism that increased the efficiency of a commercial GaP solar cell by approximately 1.1% when covered with a glass with 1% Tb^3+^ and 5% Yb^3+^ compared to undoped glass.^14^

Thus, research has been developed looking for a sensitizer ion that can effectively absorb visible solar energy and efficiently transfer it to Yb^3+^ ions. Among the lanthanide ions, the Sm^3+^ ion is a promising candidate as a sensitizer for ytterbium ions because they are rich in excited energy levels in the UV–vis region, and the large energy gap, about 7000 cm^–1^, between the excited level ^4^G_5/2_ and the lower level ^6^F_11/2_ contributes to the high emission quantum efficiency of Sm^3+^ ions.^10,11^

In this investigation, we report on the synthesis and characterization of a novel Sm^3+^/Yb^3+^ codoped oxychloride tellurite glass system. The optical properties of this material were systematically evaluated to assess its potential as a spectral converter for enhancing the efficiency of Si-based photovoltaic cells through modification of the incident solar spectrum.

## 2 Materials and Methods

The oxychloride tellurite glasses were prepared with the following chemical composition (mol %): 45TeO_2_–37BaCl_2_–18BaO, doped with 0.5SmCl_3_ and codoped with *X* % of YbCl_3_ (*X* = 0.5, 1, 2, and 3). The proportions of each reagent used are listed in Table 1. All samples were synthesized using the conventional melt-quenching method in an ambient atmosphere. The material was melted using a 95Pt/5Au crucible in a muffle furnace. The synthesis process started at room temperature with a heating rate of 13 °C/min until reaching 400 °C, where it was maintained for 1 h. Subsequently, the temperature increased from 400 to 850 °C to fully melt the mixture. The crucible was then removed from the furnace, and its bottom was placed in a container with cold water to rapidly cool the molten material. Afterward, all samples were subjected to a polishing process before characterization.

**Table 1:** Molar Concentration (%) of the Reagents Used in the Synthesis of Tellurite Samples Doped with 0.5SmCl_3_ and Codoped with YbCl_3_

sample	TeO_2_	BaCl_2_	BaO	YbCl_3_
OTGS-0	44.78	36.81	17.91	
OTGS-0.5	44.55	36.63	17.82	0.5
OTGS-1.0	44.32	36.45	17.73	1.0
OTGS-2.0	43.87	36.08	17.55	2.0
OTGS-3.0	43.43	35.70	17.37	3.0

The absorption spectra of the glasses were obtained using a PerkinElmer LAMBDA 1050 UV/vis/NIR spectrophotometer, which operates in the 175–3000 nm range. Luminescence spectra with excitation at 476 nm were obtained using an Argon laser and a spectrometer (Maya 2000, Ocean Optics). Lifetime measurements were conducted using a fluorimeter (LS55, PerkinElmer). The observed range was from 500 to 750 nm, under excitation at 476 nm. The emission and excitation slit widths were set to 5 nm. A 515 nm filter to avoid excitation scattering was used for all of the samples. The delay time was 0.2 ms. The external luminescence quantum efficiency was determined by photoacoustic spectroscopy (PAS). A Shimadzu photoacoustic cell was used, along with a xenon arc lamp from Newport, to perform the experiments. All measurements were carried out at room temperature.

## 3 Results and Discussion

The absorption coefficient spectra of the TeO_2_–BaCl_2_–BaO glass doped with SmCl_3_ and codoped with different concentrations of YbCl_3_ are shown in Figure 1. Transitions are observed in the near-infrared region, related to Sm^3+^ ions, with transitions from the ground-state ^6^H_5/2_ to higher energy states: ^6^F_1/2_ (1598 nm), ^6^H_15/2_ (1551 nm), ^6^F_3/2_ (1485 nm), ^6^F_5/2_ (1378 nm), ^6^F_7/2_ (1234 nm), and ^6^F_9/2_ (1083 nm), and in the visible region, ^6^P_3/2_ (405 nm), which are all spin allowed (Δ*S* = 0).^15,16^ The inset shows the linear dependence of the absorption coefficient value at 977 nm (^2^F_7/2_ → ^2^F_5/2_) on the nominal concentration of Yb^3+^ ions, indicating an effective incorporation of Yb^3+^ ions into the glass.

**Figure 1:**
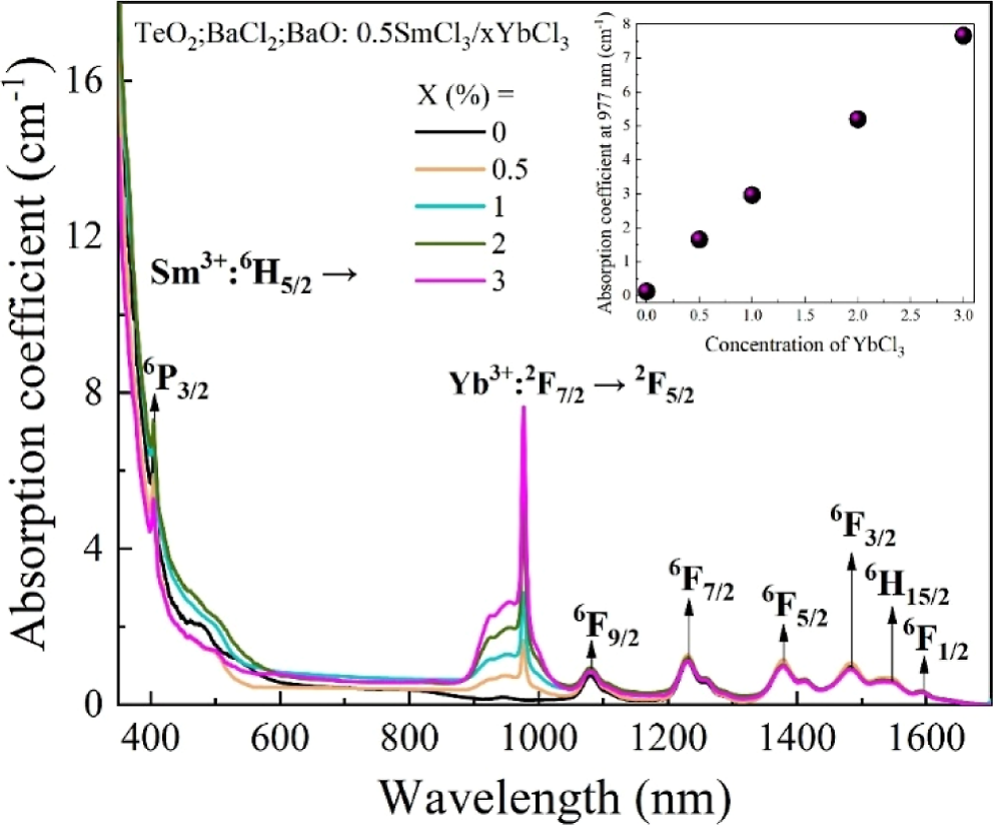
Absorption coefficient spectra of oxychloride tellurite glass codoped with SmCl_3_/YbCl_3_. The inset represents the YbCl_3_ nominal concentration dependence of the absorption at 977 nm.

Figure 2 shows the photoluminescence spectra for the samples studied under an excitation at 476 nm. Intense peaks are observed corresponding to the transitions from ^4^G_5/2_ to ^6^H_5/2_, ^6^H_7/2_, ^6^H_9/2_, ^5^H_11/2_, ^6^F_3/2_, and ^6^F_5/2_, which are centered at 564, 600, 647, 710, 908, and 953 nm, respectively, of the Sm^3+^ ions.^17^ Among the emission bands of Sm^3+^ ions, the band centered at 650 nm exhibits the highest intensity. These observed transitions are characteristic of rare-earth ions and correspond to *f*–*f* transitions.^15^ The ^4^G_5/2_ → ^6^H_9/2,11/2_ transitions are partially allowed electric dipole in nature, while the ^4^G_5/2_ → ^6^H_5/2,7/2_ transitions involve contributions from both electric and magnetic dipole transitions.^18^ By analyzing the curves for the different YbCl_3_ concentrations, it is observed that as the Yb^3+^ concentration increases, the emission intensity of Sm^3+^ ions progressively decreases. This indicates a possible energy transfer mechanism between Sm^3+^ and Yb^3+^ ions, in which the energy absorbed by Sm^3+^ is transferred to Yb^3+^. The enhancement of Yb^3+^ emission in the near-infrared region (around 980–1000 nm) is not immediately apparent in Figure 2 due to significant spectral overlap between the ^6^F_3__/__2_, ^6^F_5__/__2_ transitions of Sm^3+^ (at approximately 908 and 953 nm) and the characteristic ^2^F_5__/__2_ → ^2^F_7__/__2_ emission band of Yb^3+^. This spectral congestion makes direct visual assessment of the Yb^3+^ contribution challenging, as can be seen in the inset of Figure 2. In this context, time-resolved luminescence measurements become crucial as they provide a way to unambiguously observe and quantify the energy transfer phenomenon.

**Figure 2:**
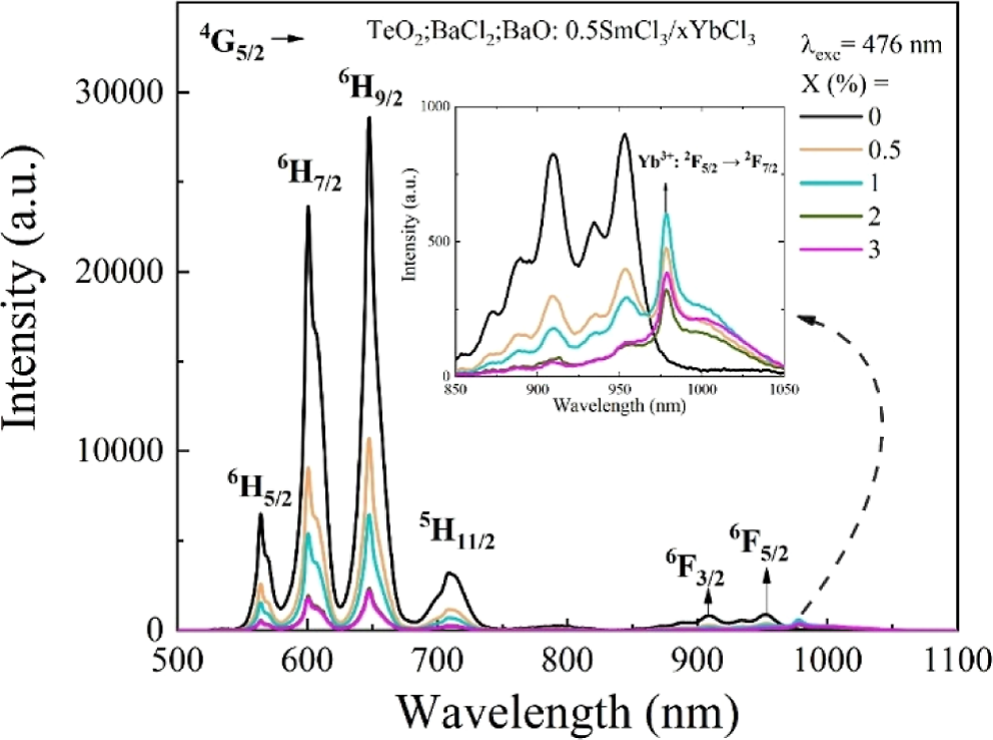
Photoluminescence of oxychloride tellurite glass codoped with SmCl_3_/YbCl_3_.

In Figure 3 are plotted the time-resolved photoluminescence spectra obtained with excitation at 476 nm for the 0.5% SmCl_3_-doped oxychloride tellurite glass. The average lifetime, ⟨τ⟩, was determined by performing the calculation of the peak area centered at 600 nm to use it as photoluminescence intensity (*I*) in the expression^19^

1 $$\left\langle \tau \right\rangle =\frac{\int I\left(t\right)tdt}{\int I\left(t\right)dt}$$

**Figure 3:**
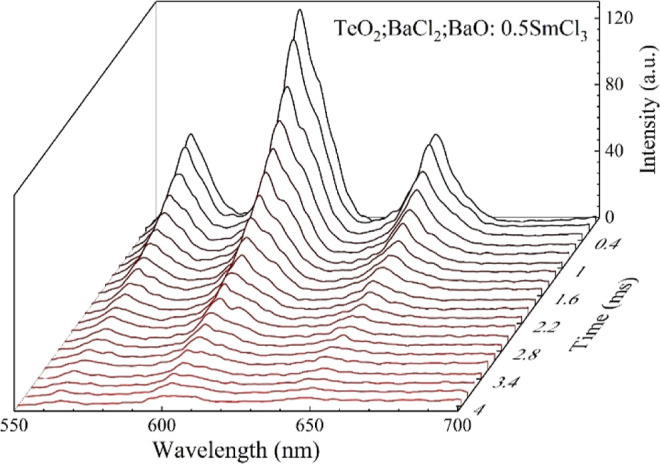
Radiative decay of the 0.5% SmCl_3_-doped oxychloride tellurite sample.

The average lifetime of Sm^3+^ progressively decreases with the increase of the Yb^3+^ concentration: for the OTGS-0 sample, τ = 0.929 ms, while sample OTGS-3.0 exhibits a reduced value of 0.226 ms, a reduction of 76% between the nondoped and the highest Yb^3+^ concentrated doped samples.

A photoacoustic-based method was employed to determine the external luminescence quantum efficiency (η_ext_).^20^ The PAS signal is directly proportional to the temperature change and the fraction of absorbed energy converted to heat within the sample. By normalizing this signal with the photoluminescence (LUM) collected simultaneously with the PAS data and assuming a single excited-state emission, the luminescence quantum efficiency can be calculated using the following equation:^20^

2 $$PAS/LUM=C\left(1-\eta_{ext}\frac{\lambda_{exc}}{\left\langle \lambda_{em}\right\rangle }\right)$$

Here, *C* is a constant dependent on the matrix, and λ_exc_ and ⟨λ_em_⟩ are the excitation and the average emission wavelengths, respectively.

Figure 4 illustrates the PAS/LUM as a function of wavelength for all of the samples analyzed in this work. Figure 4a presents the measurements conducted to determine η_ext_ for Sm^3+^ ions, corresponding to the ^4^G_5/2_ metastable state. The calculation involved dividing the slope by the intercept obtained from the linear fit, followed by multiplying the resulting ratio by the ⟨λ_em_⟩ = 647.5 nm. Consequently, a value of 57% was obtained, which is in good agreement with values reported in the literature.^21^

**Figure 4:**
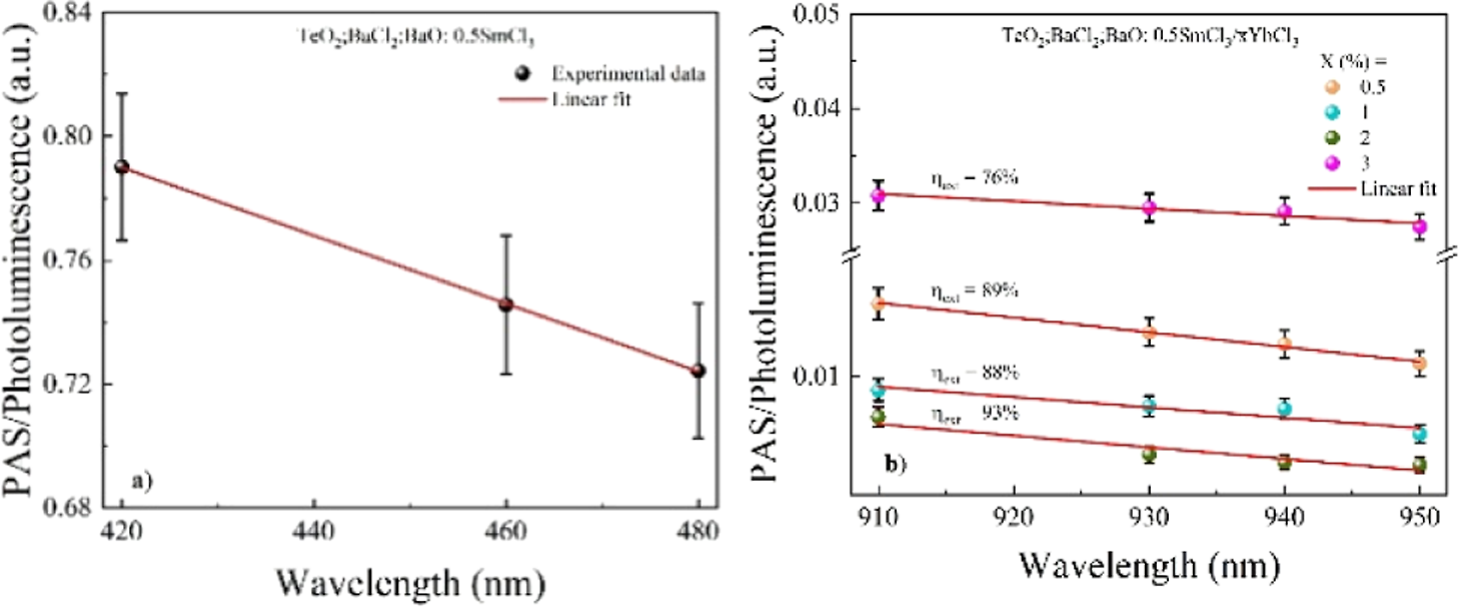
Photoacoustic signal (PAS) normalized by photoluminescence (LUM) for oxychloride tellurite glass codoped with SmCl_3_/YbCl_3_ for luminescence quantum efficiency determination.

Figure 4b presents the data obtained for the codoped samples, with measurements conducted in the Yb^3+^ absorption range. The results indicate that η_ext_ remains approximately 90% up to a concentration of 2% YbCl_3_ but decreases to 76% for the sample with the highest concentration. The calculation was performed by using ⟨λ_em_⟩ = 998 nm. All η_ext_ values, along with the corresponding lifetimes, are summarized in Table 2. A similar trend has been previously observed in tellurite glasses, and the results align well with literature values, considering an uncertainty of 10%.^22^

**Table 2:** Average Lifetime (τ), External Luminescence Quantum Efficiency (η_ext_), and Energy Transfer Efficiency (η_ET_) of Studied Glasses

sample	τ (ms)	η_ext_ (%)	η_ET_ (%)
OTGS-0	0.929	57	
OTGS-0.5	0.697	89	25
OTGS-1.0	0.597	88	36
OTGS-2.0	0.398	93	57
OTGS-3.0	0.226	76	76

The energy transfer efficiency, η_ET_, of the glass was calculated using the following equation:^23^

3 $$\eta_{ET}=\left(1-\frac{\tau_{codoped}}{\tau_{doped}}\right)\times 100\%$$

in which τ_doped_ (τ_codoped_) corresponds to the average lifetime measured for the doped (codoped) samples. Both the experimental lifetime values of the ^4^G_5/2_ excited state and the energy transfer efficiency of our oxychloride tellurite samples are displayed in Table 2. Hence, η_ET_ increases as the Yb^3+^ concentration increases, going from 25% to 76% for samples OTGS-0.5 and OTGS-3.0, respectively. The outcomes indicate that Sm^3+^ acts effectively as a sensitizer, while Yb^3+^ serves as an activator.

Other works also reported energy transfer between Sm^3+^ and Yb^3+^ ions in different matrixes. Herrera et al. (2021) investigated a GeO_2_–PbO glass matrix codoped with Sm^3+^/Yb^3+^ ions. Their findings revealed that specimens containing 1 mol % of Sm_2_O_3_ and 0.5 mol % of Yb_2_O_3_ exhibited a significant reduction in the Sm^3+^ luminescence lifetime, which was attributed to an energy transfer mechanism between Sm^3+^ and Yb^3+^ ions. The η_ET_ was quantified as 23.8%, with the process being ascribed to a cross-relaxation mechanism.^24^ Xia et al. (2017) studied a phosphorus of SrMoO_4_ codoped with 2 mol % of Sm_2_O_3_ and 1 mol % of Yb_2_O_3_ and observed an increase in the emission intensity in the near-infrared, with an energy transfer of approximately 36.5%.^25^ Furthermore, investigations with the TeO_2_–ZnO–Nb_2_O_5_–TiO_2_ glass codoped with 1 mol % Sm_2_O_3_ and 3 mol % Yb_2_O_3_ demonstrated a comparable reduction in luminescence lifetime, achieving a notably higher η_ET_ of 88%.^26^

The energy transfer between Sm^3+^ and Yb^3+^ ions is supported by the increase in emission in the near-infrared region when Yb is added into the glass, which is attributed to the luminescence of Yb^3+^ ions, as observed in Figure 2, but mainly by the decrease observed in the lifetime of the ^4^G_5/2_ state. Figure 5 shows a partial energy level diagram to illustrate the cross-relaxation energy transfer mechanism involved in the Sm^3+^ and Yb^3+^ ions. Upon blue photoexcitation, Sm^3+^ ions undergo electronic transition to the metastable (^4^G_5/2_) excited state, from where they may decay radiatively or nonradiatively to different lower states. However, the energy absorbed by Sm^3+^ can also be transferred to an activator, Yb^3+^, through a cross-relaxation (CR) process between ^4^G_5/2_ → ^6^F_7/2_ and ^2^F_7/2_ → ^2^F_5/2_ states.^5^ This channel should allow the donation of energy from only one Sm^3+^ ion to a single Yb^3+^ ion.

**Figure 5:**
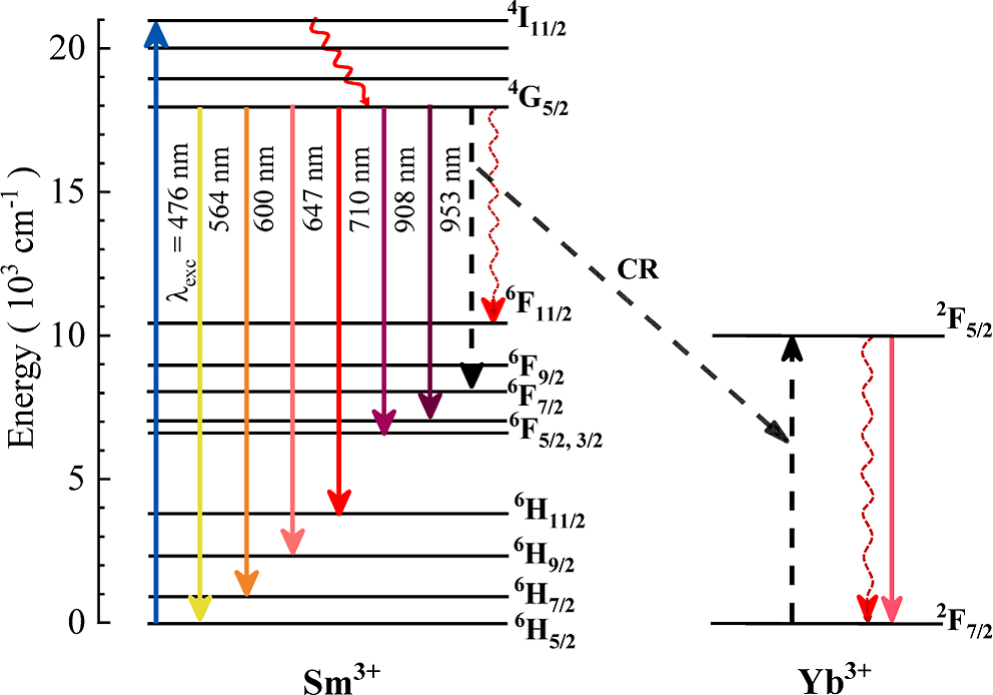
Energy level diagram of Sm^3+^ and Yb^3+^ ions. The dashed arrows represent cross-relaxation and potential energy transfer mechanisms.

Finally, when evaluating a new material as an energy converter, it is standard practice to calculate the total quantum efficiency, which is defined by the following equation:^23^

4 $$\eta_{QE}=\eta_{Sm}\left(1-\eta_{ET}\right)+2\eta_{Yb}\eta_{ET}$$

Here, η_Sm_ and η_Yb_ represent the luminescence quantum efficiencies of Sm^3+^ (OTGS-0, η_ext_) and Yb^3+^ (from OTGS-0.5 to OTGS-3.0, η_ext_) as listed in Table 2. For the sample with the highest concentration in this study, a total quantum efficiency of η_QE_ = 129% was calculated.

## 4 Conclusions

The optical spectroscopy investigation shows a good incorporation of Sm^3+^ and Yb^3+^ ions into the oxychloride tellurite glass matrix. The Yb^3+^ ions exhibited an approximately linear behavior with the YbCl_3_ concentration increase in the host. The time-resolved luminescence results indicate that an efficient energy transfer mechanism occurs as the Yb^3+^ concentration is increased in the material, which leads to a decrease in the average lifetime of the metastable state of Sm^3+^. A high value of 76% for energy transfer efficiency was determined for the sample with 3% YbCl_3_, and the most likely channel for this transfer is a cross-relaxation process from the ^4^G_5/2_ → ^6^F_7/2_ to the ^2^F_7/2_ → ^2^F_5/2_ state, as evidenced by the ^4^G_5/2_ lifetime values. The observation indicates that the oxychloride tellurite glass holds promise for energy conversion applications, from the visible to the near-infrared region, where Si solar cells have a better response.
